# Pharmacological activation of the hERG K^+^ channel for the management of the long QT syndrome: A review

**DOI:** 10.1002/joa3.12741

**Published:** 2022-06-14

**Authors:** Aziza El Harchi, Oriane Brincourt

**Affiliations:** ^1^ School of Physiology, Pharmacology and Neuroscience, Biomedical Sciences Building University of Bristol, University Walk Bristol UK

**Keywords:** Long QT syndrome, hERG K^+^ channel pharmacology, mutation, arrhythmia, hERG agonists

## Abstract

In the human heart, the rapid delayed rectifier K^+^ current (*I*_Kr_) contributes significantly to ventricular action potential (AP) repolarization and to set the duration of the QT interval of the surface electrocardiogram (ECG). The pore‐forming (α) subunit of the *I*_Kr_ channel is encoded by *KCNH2* or human ether‐à‐go‐go‐related gene 1 (hERG1). Impairment of hERG function through either gene mutation (congenital) or pharmacological blockade by diverse drugs in clinical use (acquired) can cause a prolongation of the AP duration (APD) reflected onto the surface ECG as a prolonged QT interval or Long QT Syndrome (LQTS). LQTS can increase the risk of triggered activity of ventricular cardiomyocytes and associated life‐threatening arrhythmia. Current treatments all focus on reducing the incidence of arrhythmia or terminating it after its onset but there is to date no prophylactic treatment for the pharmacological management of LQTS. A new class of hERG modulators (agonists) have been suggested through direct interaction with the hERG channel to shorten the action potential duration (APD) and/or increase the postrepolarisation refractoriness period (PRRP) of ventricular cardiomyocytes protecting thereby against triggered activity and associated arrhythmia. Although promising drug candidates, there remain major obstacles to their clinical development. The aim of this review is to summarize the latest advances as well as the limitations of this proposed pharmacotherapy.

## Graphical abstract

Pharmacological activation of the hERG potassium channel for the management of the Long QT Syndrome (LQTS) is a promising strategy. There are however still major drawbacks to its pre‐clinical development. This review will highlight the benefits as well as limitations of the proposed strategy for the management of LQTS.

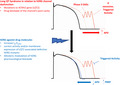

## 1 INTRODUCTION

### 1.1 Congenital and acquired long QT syndrome

**FIGURE 1:**
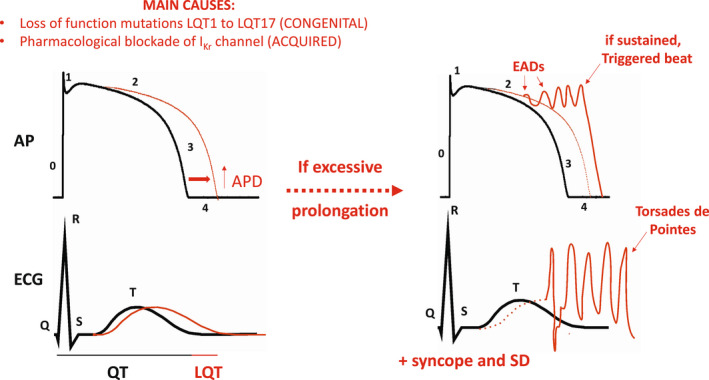
Diagram linking main causes of LQTS and associated arrhythmia with their effects on biological markers of the human cardiac ventricular repolarization. Left: LQTS is a cardiac disorder either due to mutations to genes encoding key ionic conductances of the cardiac AP or to pharmacological blockade of the *I*_Kr_ channel causing delayed repolarization (↑APD; red) (upper panel). This delay translates onto the ECG as a prolongation of the QT interval (LQT; red) (lower panel). Right: When in excess, delayed repolarization can favor at the cellular level the reopening of Ca^2+^ and/or Na^+^ channels. These outward currents generate early afterdepolarizations (EADs; red) that are depolarizing oscillations of the membrane potential of ventricular cardiomyocytes (upper panel). The triggered activity arising from EADs increases the risk of the life‐threatening arrhythmia Torsades de Pointes (TdP; red) arrhythmia that can degenerate into ventricular fibrillation (lower panel). LQTS patients can also experience syncope and sudden death.

Long QT syndrome (LQTS) is a relatively rare and potentially fatal cardiac disorder characterized by a prolongation of the QT interval of the surface electrocardiogram (ECG) and T‐wave abnormalities.^1^ Lengthening of the QT interval is due to a delay in the repolarization phase of the action potential (AP) of ventricular cardiomyocytes (Figure 1). Delayed repolarization can favor the development of early afterdepolarizations (EADs) (Figure 1), oscillating depolarizations of the membrane potential of ventricular cardiomyocytes emerging during phase 2 or 3 of the action potential (Figure 1).^1^, ^2^ EADs are thought to result mainly from the reopening of L‐type calcium channels (*I*_Ca,L_)^1^, ^2^ and be facilitated by the increase in the late component of the cardiac sodium current (*I*_Na,L_).^3^ They constitute a major pro‐arrhythmic risk factor for the generation of the potentially fatal arrhythmia Torsades de Pointes (TdP)^1^, ^2^ with the risk of degeneration into ventricular fibrillation (VF) and sudden cardiac death (SCD)^1^, ^2^ (Figure 1).

LQTS can be inherited, and mutations in 17 genes coding α or β and accessory subunits of major ion channels of the cardiac AP have been associated with numerous familial cases of LQTS to date.^4^, ^5^ There are currently three predominant forms of congenital LQTS (cLQTS), with mutations to genes *KCNQ1* (LQT1), *KCNH2* (LQT2), and *SCN5A* (LQT3), accounting for 75% of all genotype positive cases.^4^ Each of these genes encodes particularly pore‐forming (α) subunits of key ionic channels of the cardiac action potential. Thus, *KCNQ1* encodes the Kv7.1 channel giving rise to the slow‐delayed rectifier K^+^ current *I*_Ks,_ ^4^, ^5^ which along with the rapid delayed rectifier K^+^ current *I*_Kr_ (Kv11.1 protein channel; *KCNH2* gene also known as *hERG1* gene) control the duration of the repolarization phase of the cardiac AP.^4^, ^5^, ^6^, ^7^ The *SCN5A* gene encodes the Nav1.5 sodium channel underlying I_Na_ that mainly governs the initial depolarization phase of the AP.^4^, ^5^, ^7^

The syndrome can also be acquired due to electrolyte imbalance, medical conditions such as thyroid disease, or most commonly due to pharmacological inhibition of the pore‐forming α‐subunit of the *I*_Kr_/*I*_hERG_ channel by commonly used medications (also known as the drug‐induced long QT syndrome, diLQTS, or acquired long QT syndrome, aLQTS).^2^, ^6^, ^7^ Pharmacological blockade of the *I*_Kr_/*I*_hERG_ channel is a major issue for drug development, and although the incidence of TdP with specific drugs is low, the strong link between the *I*_Kr_ channel and TdP requires that all new pharmaceutical agents must be screened against this channel as part of preclinical assessments of cardiac safety.^2^, ^6^ [Correction added on June 22, 2020, after first Online publication: "This paragraph was duplicated, hence it has been removed.]

### 1.2 Current clinical management of LQTS and use of hERG agonist drug molecules as a potential new therapy

The first‐choice pharmacotherapy for symptomatic congenital LQTS patients is β‐adrenergic receptor blockade in an attempt to reduce the occurrence of cardiac events.^1^, ^4^, ^5^, ^8^ However, limitations to the efficacy of blockade remain, with syncope, aborted cardiac arrest, and LQTS‐related death reported in patients receiving β‐blocker therapy, particularly in cLQT2 patients.^8^ Treatment for the acquired form, secondary to pharmacological hERG blockade, is largely supportive. First‐line treatment consists mainly of the withdrawal of the culprit drug and/or managing electrolyte imbalances in order to restore ventricular action potential duration (APD) to within the physiological range and thereby mitigate against the risk of TdP and associated arrhythmia.^7^

For both forms of LQTS, if ongoing arrhythmic risk remains sufficiently high, implantation of an implantable cardioverter defibrillator (ICD) is indicated,^4^, ^9^ with the associated risk of complications this brings.^9^ There is in fact a significant burden of morbidity and mortality seen as a result of procedural complications, infection, as well as the ongoing implications on quality of life and the risk of inappropriate shocks.^9^ Additionally, the young age of many patients receiving ICD therapy for LQTS increases the likelihood of device‐related complications during the patient's lifetime.^9^ Current treatment for the management of LQTS, therefore, focuses on either reducing the incidence of arrhythmia triggers by careful management of electrolytes and concurrent medication or terminating the arrhythmia after onset (ICD implantation). To date, however, there is no effective or evidence‐based treatment for preventing the onset of TdP in the first place, which could be useful for the management in patients of recurrent cardiac arrhythmia associated either with cLQTS or long‐term treatment therapies with QT‐prolonging agents.

Various pharmacotherapeutic options have been evaluated. Thus, the off‐label use of sodium channel blockers such as mexiletine or ranolazine for the genetic‐specific therapy of LQT3^10^ has been suggested in the 2015 guidelines of the European Society of Cardiology (ESC) as an effective clinical practice.^11^ These agents are thought to reduce the risk of triggered activity through their reported inhibitory effects on *I*_Na,L._ ^10^, ^11^ Although not directly targeting the underlying mechanism of the disease (i.e., defective hERG activity), inhibitors of *I*_Na,L_ have also been suggested to be effective for the treatment of LQT2^10^; however, clinical investigations to demonstrate their efficacy and safety are still ongoing.^12^ The late sodium blockers mexiletine and lidocaine have also been suggested for the management of diLQTS,^13^, ^14^, ^15^ especially in cases refractory to conventional interventions where removal of culprit drug and or ICD implantation is undesirable or contraindicated.^13^, ^14^ Another proposed target has been the selective activation of *I*_Kr_/*I*_hERG_ by small activator drug molecules (hERG agonists) to increase repolarization reserve and counteract LQTS‐associated triggered activity. This potential mechanism‐based therapy has been investigated in several in vitro, in vivo, and in silico studies^16^, ^17^, ^18^, ^19^, ^20^, ^21^, ^22^, ^23^, ^24^, ^25^, ^26^, ^27^, ^28^, ^29^, ^30^, ^31^, ^32^ for the management of the effects of both LQT2‐associated mutations to *hERG1* ^20^, ^24^, ^25^, ^26^, ^27^, ^28^ and/or pharmacological blockade of the hERG channel.^16^, ^17^, ^18^, ^19^, ^20^, ^21^, ^23^, ^31^ Although promising drug candidates, there are still major drawbacks to their preclinical development and consequently none of these drug molecules have to date been trialled in clinical settings. This review will therefore focus on the antiarrhythmic benefit of some of the most effective hERG activators identified to date from various in vitro, in vivo, and in silico experimental models of LQTS. It will also highlight the limitations of the proposed strategy.

## 2 THE *I*_Kr_ / *I*_hERG_ CHANNEL STRUCTURE FUNCTION IN RELATION TO CARDIAC REPOLARIZATION AND LQTS

**FIGURE 2:**
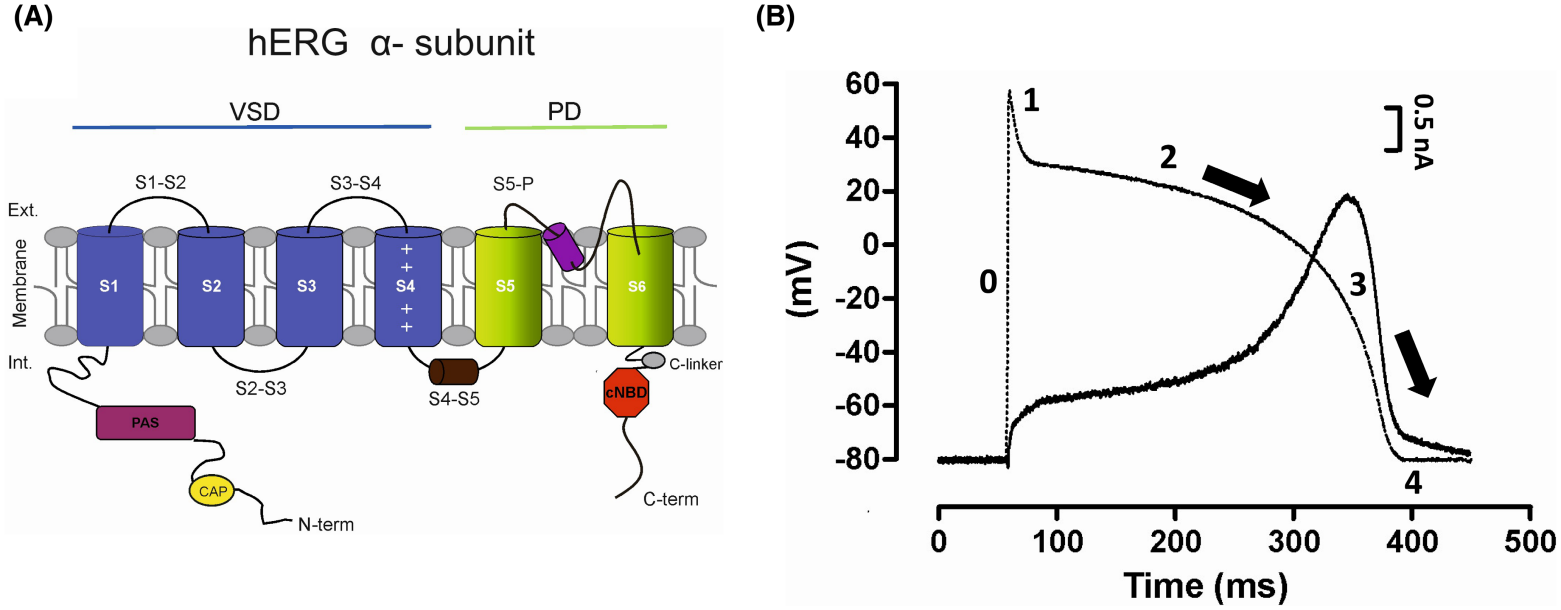
(A) Schema of a single α‐subunit of the hERG channel. Structure of the hERG α‐subunit comprises six helical transmembrane segments (S1–S6). The α‐helices S1 to S4 constitute the voltage‐sensing domain (VSD); while α‐helices S5 and S6 (in green) delimit the pore domain (PD). Distinctive structures important to hERG function are a short S4–S5 linker and an intracellular N‐terminus containing a PAS domain (purple) and a short CAP domain (yellow). The isoform hERG1b (not shown) exhibits a shorter N‐term which is thought to underlie in part the differential biophysical properties between both isoforms. Also of importance is the C‐terminus containing a cNBHD domain (red) coupled to the pore by a C‐linker (grey). (B) Time course of *I*_Kr_/*I*_hERG_ (solid black line) during the course of a mathematically modeled human ventricular action potential (superimposed in dashed line). Representative time course of *I*_hERG_ recorded at 37°C during a ventricular AP voltage command from hEK‐293B cells stably expressing WT hERG1a. Due to its fast inactivation, *I*_Kr_/*I*_hERG_ amplitude is small throughout the duration of the plateau phase (phase 2) of the action potential. As depolarization progresses *I*_Kr_/*I*_hERG_ increases to then peak during phase 3 of the AP giving rise to “resurgent *I*_Kr_/*I*_hERG_.” Due to its slow deactivation, *I*_Kr_/*I*_hERG_ produces some repolarizing current (“diastolic” *I*_Kr_/*I*_hERG_) sometimes after completion of the AP (phase 4). Black arrows indicate the direction of repolarization.

In the human heart, the rapid delayed rectifier K^+^ current (*I*_Kr_/*I*_hERG_) contributes significantly to ventricular AP repolarization and to set the duration of the QT interval of the surface ECG.^1^, ^6^ The pore‐forming α‐subunit of the hERG channel has six transmembrane domains (6; see also Figure 2A) comprising common structures with other Kv channels as well as unique features (^6^; see also Figure 2A). Four α‐subunit assemble in tetramers to produce functional channels.^6^ Two main alternate transcripts of *hERG1* (hERG1a and hERG1b) are thought to coassemble to form functional sarcolemmal *I*_Kr_ channel tetramers proteins with the hERG1b isoform possessing a shorter, distinct N‐terminus.^6^, ^33^ It has been suggested that hERG may coassemble with β accessory subunits of the KCNE family contributing thus to native *I*_Kr_ (for review in ^6^), including coassembly with MinK‐related peptide 1 (MiRP1; *KCNE2* gene) or the regulatory β‐subunit MinK (*KCNE1* gene).^6^ Figure 2B shows the contribution of *I*_Kr_/*I*_hERG_ during the time course of a ventricular AP. Due to its fast inactivation, *I*_Kr_/*I*_hERG_ contributes minimally to the ventricular AP (Figure 2B). However, as depolarization progresses, *I*_Kr_/*I*_hERG_ amplitude increases throughout early repolarization during the AP plateau thus giving rise to hERG resurgent current. *I*_Kr_ declines during terminal repolarization but its slow deactivation allows for some repolarizing current to flow after the completion of the action potential during the diastolic interval (Figure 2B). Consequently, *I*_Kr_ can act to offset EADs, potentially arrhythmogenic premature depolarizations at the start of the diastolic interval.^6^, ^34^

More than 500 loss of function mutations within *hERG1* have been linked with LQT2 located in various regions of its α‐subunit.^4^, ^35^ It is the second most common subtype affecting 25–30% of LQTS individuals^4^, ^5^ with most LQT2‐linked mutations being missense mutations that cause the misfolding and result in the retention of the channel complex within the endoplasmic reticulum (ER) of Kv11.1 proteins.^35^ Retention within the ER impairs the trafficking of the hERG channel to the cell membrane thereby causing a reduction in *I*_Kr_/*I*_hERG._ ^4^, ^35^ To a lesser extent, some of this loss of function mutations (<10% of all LQT2 mutations) can reduce *I*_Kr_/*I*_hERG_ through impairment of the hERG channel gating or K^+^ permeation.^35^ Some LQTS mutations have also been located in the *KCNE2* gene (LQT6),^4^, ^5^, ^36^ but they are by comparison rare variants (<0.1% of all LQTS individuals)^5^, ^36^ and are thought to have limited clinical significance in the absence of additional predisposing factors.^36^

The strong link between hERG channel dysfunction, diLQTS, and associated TdP lies in hERG's unique structural features of its pore inner cavity. First, it lacks the highly conserved pro‐X‐pro‐motif that, in other voltage‐gated potassium channels, reduces the volume of the pore inner cavity.^2^, ^6^, ^37^ Secondly, its pore contains specific aromatic amino acid residues (tyrosine at 652 and phenylalanine at 656; Y652 and F656) in the S6 helices of the channel,^2^, ^6^, ^37^ with deep hydrophobic pockets surrounding the central cavity, suggested to contribute to the channel's unusual sensitivity to diverse drugs, as identified in a recent cryo‐EM modeling study (^38^; for review^39^). Further, many pharmacological agents may also be contingent to varying extents upon the channel's gating kinetics to exert their inhibitory effects (reviewed in^2^, ^6^, ^37^). Thus, strong experimental evidence supports the notion that high‐affinity drug blockade of the hERG channel may be strongly dependent on the conformational changes associated with inactivation gating. In this scheme (updated in great detail in the new cryo‐EM structure of the hERG channel in^39^), the inactivation‐dependent conformation changes would optimize drug inhibitor molecules' interaction with S6 and/or pore helix residues of the inner cavity thus facilitating their binding and/or retention in the channel inner cavity.^2^, ^6^, ^37^, ^39^ Some hERG inhibitors have also been reported to demonstrate a tendency to become trapped within the channel's central cavity upon closure of the channel's activation gate^2^, ^6^, ^37^; another mechanism accounting for hERG's unique tropism for drugs that may increase pro‐arrhythmic risk (for review ^2^, ^6^, ^37^).

## 3 CLASSIFICATION OF hERG AGONISTS

**FIGURE 3:**
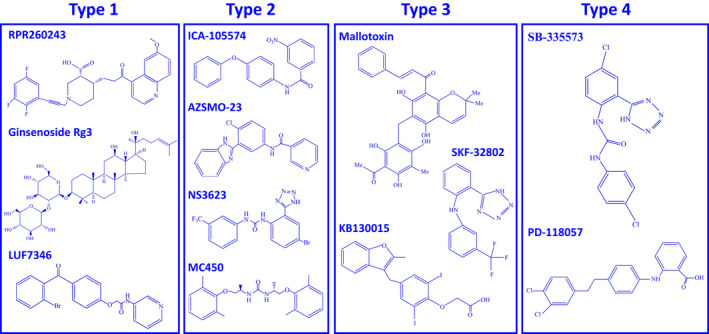
Molecular graphical representations of type 1–4 hERG activator drug molecules. Type 1–4 activators exhibit great structural diversity. All chemical structures were drawn as two‐dimensional structures using the chemical structure drawing program ChemDraw®.

**FIGURE 4:**
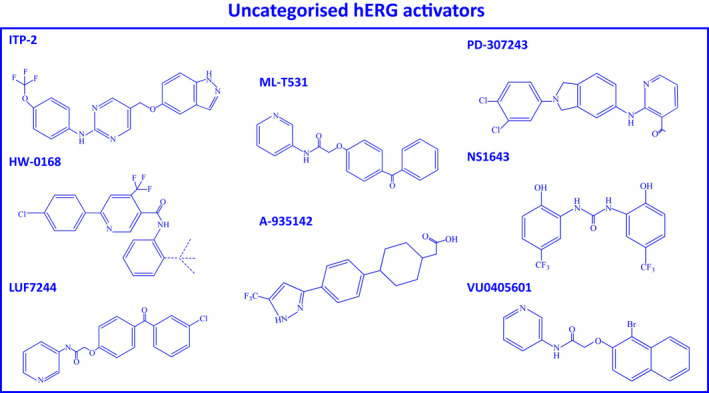
Molecular graphical representations of uncategorized hERG activator drug molecules. All chemical structures were drawn as two‐dimensional structures using the chemical structure drawing program ChemDraw®.

To date, 20 drug agonist molecules (activators) of the hERG channel have been identified and categorized into four different types depending on their main reported mechanisms of action (40; see also Figure 3). Some, however, have also been reported to exhibit multiple mechanisms of actions (^40^, ^41^; see also Figure 4). These pharmacological agents display great structural diversity (see Figures 3, 4). They also exhibit complex biophysical interactions with the hERG channel and enhance *I*_hERG_ amplitude through various mechanisms (see Tables 1, 2, 3 for their various properties and/or effects). Tables 1, 2, 3 list all hERG agonist molecules reported to date along with an indication of their molecular determinants of actions if mapped, effects on other cardiac and noncardiac ion channels, antiarrhythmic benefit against the effects of congenital and/or acquired LQTS, and associated pro‐arrhythmic risk. Agonists exhibiting a type 1 mechanism primarily enhance hERG current by slowing the closure of the channel's activation gate (deactivation) but may also cause a modest reduction in the channel's ability to inactivate (Table 1). Type 2 agonists (see Table 1) act mainly through a rightward shift in the voltage dependence of inactivation, whereas type 3 (Table 2) and type 4 agonists (Table 2) negatively shift the voltage dependence of activation (also known as “facilitators”) or increase the open probability of the channel (type 4 also known as “pore modifiers”), respectively. Also included is an additional table (Table 3) listing all activators that may combine properties from up to three different classes and/or that are yet to be formerly categorized either because of the lack of thorough investigation of their biophysical effects on the hERG channel (e.g., LUF7244), they may exhibit features of their own (e.g., ITP‐2) or because of the interlaboratory variability in the characterization of their biophysical effects.

**TABLE 1:** *Type 1 activators* act mainly through a reduction in the rates of the hERG channel deactivation and modest attenuation of inactivation gating. *Type 2 activators* attenuate C‐type inactivation through a dual mechanism involving either a shift in the voltage dependence of inactivation to more depolarized membrane potentials and/or a slowing of the rate of inactivation onset

Name	Type	Molecular determinants of activity	Channel selectivity	Reported benefit for LQTS	Reported associated Pro‐arrhythmic risk	Ref.
RPR260243	1	Residues in the intracellular ends of the S5 helix (L553, F557) and an adjacent region of the S6 helix (N658, V659) of a single hERG subunit. Also, strong hydrophobic bonding interactions with M645.	No effect on recombinant cardiac channels hNav1.5 (I_Na_)_,_ hKCNQ ‐KCNE1 (I_Ks_) Weakly inhibits cardiac I_Ca,L_ recorded from guinea pig cardiomyocytes. It inhibits the recombinant neuronal erg3 channel. Differential sensitivity for recombinant hERG1a and hERG1b.	diLQTS: corrects for electrophysiological effects of pharmacologically inhibited hERG currents in guinea pig cardiomyocytes and zebrafish hearts cLQTS: corrects for effects of trafficking deficient LQT2‐associated R56Q hERG.	Possible risk associated with impairment of conduction velocity in guinea pig hearts (prolonged PR interval of the ECG)	18, 23, 26, 41, 49, 70, 73
Ginsenoside RG3	1	Residues in the S1 (Y420), S2 (L452, F463, and S4 (I521, K525).	No report	diLQTS: reduces effects of antithyroid cancer drug‐vandetanib induced LQTS in hiPSCMs.	No report	74, 75
LUF7346	1	No report	No measured effect on cardiac I_Ks_, I_Ca,L_ in hiPSCMs and possibly negligible effect on I_K1_ and I_Na_ as no change induced to AP amplitude and diastolic potential of hiPSCMs	diLQTS: rescues pharmacologically induced LQTS in WT, JLNS, and LQT1‐associated mutations in hiPSCMs. cLQTS: Corrects for electrophysiological effects of LQT2‐N996 hERG, LQT1‐R190Q, and JLNS‐R594Q KCNQ1.	No report	^20^
ICA‐105574	2	Residues in a hydrophobic pocket in the pore between two adjacent subunits. Interacting residues (F557, T623, Y652, and F656) are located in the pore helix, based on the selectivity filter and S6 segment. Mutation to Y652, F557, L622, and F656 reduce ICA activity. Mutation to M645 (M645C) accelerates the ICA‐mediated rate of inactivation.	Suggested absence of effect on cardiac I_K1_ and I_Na_ as no change induced to AP amplitude and diastolic potential of guinea pig cardiomyocytes.	diLQTS: reduces electrophysiological effects associated with hERG inhibition by various drug inhibitors (e.g., high‐affinity E‐4031 dofetilide, low‐affinity moxifloxacin). It did, however, not change the potency of the external blocker and toxin BeKm‐1. cLQTS: ICA‐105574 could activate both the LQT2‐associated hERG mutants N470D and G601S but with threefold reduced potency compared with WT. Effective against the variant of uncertain significance (VUS) KCNH2 T983I.	Overcorrection of the APD to the point of triggering ventricular fibrillation at high concentrations. Recapitulates the electrophysiologic and arrhythmic manifestations of SQT1 by creating the substrate for reentry.	16, 24, 28, 30, 41, 45, 53, 58, 76
AZSMO‐23	2	Behaves as a blocker when Y652A mutation occurs. Activator activity enhanced against F656T	Inhibits recombinant channels hNav1.5 (I_Na_), hKCNQ1‐hKCNE1 (I_Ks_) hKv4.3‐hKChIP2.2 (I_to_), Cav3.2 (I_Ca,T_), and Kv1.5 (I_Kur_) channels. It activates hCav1.2/β2/α2δ (I_Ca,L_) channels. No effect on hHCN4 (*I*_f_).	No report	No report	^77^
NS3623	2	F656M as well as S620T and S631A exhibit enhanced agonist activity.	In cultured canine cardiomyocytes, it has no effect on I_K1_ but increases I_to_ in epicardial and midmyocardial cardiomyocytes, which as a result enhances I_Ca,L_. No effect on I_Kur_, I_Ca,T_, and I_Na_	diLQTS: reverses E‐4031 induced QT prolongation in anesthetized and conscious guinea pigs.	Possible risk associated with impairment of conduction velocity in guinea pig hearts (prolonged QRS interval) and activation of native I_Kr_ in the sinus node and vagal fibers.	41, 46, 65, 66, 78
MC450	2	No report	No report	No report	No report	^79^

**TABLE 2:** *Type 3 activators* induce a shift in the voltage dependence of activation of the hERG channel to more hyperpolarized membrane potentials. *Type 4 activators* act mainly by increasing the channel's open probability (also known as “pore modifiers”)

Name	Type	Molecular determinants of activity	Channel selectivity	Reported benefit for LQTS	Reported associated Pro‐arrhythmic risk	References
SKF‐32802	3	Strong interaction with the selectivity filter (SF). Hydrogen bond with T623. Has an equal affinity for the opened and closed states of the channel.	Weak blocker of recombinant hNav1.5 (I_Na_) and hCav1.2 (I_Ca,L_) channels.	diLQTS: concentration‐dependent right‐shift of the pIC_50_ curves of quinidine.	No report	^41^
Mallotoxin (MTX)	3	No report	Activates Ca^2+^‐activated K^+^ (BK channel) at 0.5 μM.	No report	Shortens QT interval, JT interval, increases Tp‐Te, and rTp‐Te at 0.1 μm, elicits ventricular fibrillation (VF) at 1 μM in isolated rabbit hearts.	61, 80
KB130015	3	Acts from the intracellular side and presumably binds to the hERG pore from the cytosolic side. Y652 may be part of the binding site important for channel opening by KB130015.	It inhibits I_KAch_ from ventricular guinea pig myocytes. Inhibits I_Ca,L_ and I_KATP_ channels. Slows the inactivation of voltage‐dependent Na^+^ channels. Activates I_Ks_ and large‐conductance calcium‐activated potassium (BK) channels. No effect on I_K1_ and I_to_.	diLQTS: Functionally competes with hERG block by amiodarone and E‐4031.	No report	41, 55, 81
SB‐335573	4	Strong interaction with the selectivity filter (SF). Tetrazole nitrogen accepts a weak hydrogen bond from the side‐chain ‐OH on S624. Makes hydrogen bonds to S649 and M645 on an adjacent subunit. Has an equal affinity for the opened and closed states of the channel.	Weak blocker of recombinant hNav1.5 (I_Na_) and hCav1.2 (I_Ca,L_) channels.	diLQTS: ineffective in rightshifting quinidine pIC_50_ curve but did reduce the effect at the single concentrations of 0.37, 1.11, and 3.33 μM in a concentration‐dependent manner.	No report	^41^
PD‐118057	4	Molecular modeling indicates that PD‐118057 binds to a hydrophobic pocket formed by L646 in the S6 domain and L622 and F619 of an adjacent subunit. Mutation to F619 and L646 suppresses agonist activity. Mutation to C643 and M645 enhances drug activity.	No effect on I_Na_, I_Ca‐L_, I_K1_, and I_Ks_ recorded from isolated guinea pig cardiomyocytes.	diLQTS: 3 μM prevents APD, EADs, and QT prolongation caused by high‐affinity inhibitor dofetilide. cLQTS: Failed to rescue trafficking defective LQT2‐associated E637K hERG mutant.	Increases arrhythmia provocations in perfused canine atrial preparations with a combination of ERP abbreviation and TDR amplification. Recapitulates the electrophysiological and arrhythmic manifestations of SQT1 by creating the substrate for reentry.	22, 41, 52, 53, 59, 60

**TABLE 3:** Activators with suggested multiple mechanisms of actions onto the hERG channel

Name	Type	Molecular determinants of activity	Channel selectivity	Reported benefit for LQT	Reported associated pro‐arrhythmic risk	References
ITP‐2	2, 3	May act from the extracellular side of the membrane.	No report	cLQTS: 3 μM ITP‐2 reported to activate trafficking deficient LQT2‐associated G601S with twofold reduced potency, compared with the WT but reported to fail to activate N470D.	No report	^28^
HW‐0168	2, 3 but very limited potentially type 4	No report	No report	No report	Possible risk of overcorrection of the APD recorded from isolated guinea pig ventricular cardiomyocytes.	^82^
LUF7244	1, 2	Interacts with F557 (S5), F619 (P‐Helix), and Y652 (S6). May have close contact with SF residue T623 and S649 (S6).	No effect at 10 μM on recombinant hKir2.1 (I_K1_) and hNav1.5 (I_Na_) channels. No effect on I_Ca‐L_ and I_Ks_ recorded from canine ventricular cardiomyocytes.	diLQTS: Negative effect on the binding of a series of blockers (astemizole, sertindole, dofetilide, and cisapride) by an allosteric mechanism. Decreases dofetilide‐induced AP lengthening and EADs in human and canine cardiomyocytes in vitro. cLQTS: In G601S cells, dofetilide + LUF7244 treatment increases I_Kr_.	Possible risk of overcorrection as shortens APD by 50% in hiPSCMs and canine cardiomyocytes.	17, 19, 25
ML‐T531	1, 2	No report	No effect on recombinant hCav1.2 (I_Ca‐L_), hKir2.1 (I_K1_), hNav1.5 (I_Na_), and hKv4.3 (I_to_) channels. At 10 μM has a minor suppressive effect on hKCNQ1‐KCNE1 channels (I_Ks_).	diLQTS: Negative effect on the binding of blockers (astemizole, sertindole, dofetilide, and cisapride) by an allosteric mechanism. cLQTS: I_Kr_ increase in native human cardiomyocytes from LQT1 patients.	No report	19, 29
A‐935142	1, 2, and possibly 3	Strong aromatic interactions at Y652. Polar bonding interactions at S624 increase pore open probability. The binding site is likely not to overlap that of typical hERG blockers.	No report	diLQTS: did not prevent inhibition of the hERG channel by low‐affinity inhibitors sotalol and terfenadine suggesting it may be ineffective against the effects of diLQTs. This experimental evidence warrants further investigation.	No report	41, 54
PD‐307243	2, 4	Works from the extracellular side of the cell membrane as it acts on the pore loop. Hydrogen‐bonding interactions at S624 may be responsible for the increase in the pore‐opening probability.	Activates I_Ca,L_ but no effect on I_to_ recorded from ventricular cardiomyocytes isolated from rabbit hearts. No effect on recombinant hKCNQ1‐KCNE1 (I_Ks_) and hNav1.5 (I_Na_) channels.	diLQTS: Dofetilide prevents the activator effect of PD and unmasks its current suppressing effect. In presence of BeKm‐1, PD's activator effect was potentiated. Altogether, this experimental evidence raises caution and warrants further investigation.	No report	41, 83
NS1643	1, 2, and possibly 3	Work from the extracellular side of the cell membrane as it binds to the outer vestibule/pore entrance of hERG. Three possible binding sites in the vicinity of L529 for the open state. Agonist activity facilitated by mutations (F656 to Val, Met, or Thr). Hydrogen‐bonding interactions with M645 and S624. Hydrophobic interactions (M554, F557) and aromatic interactions (F619).	Suggested absence on cardiac I_K1_ and I_Na_ as no change induced to AP amplitude and diastolic potential of guinea pig cardiomyocytes. Inhibitor of neuronal Kv12.1 ether‐à‐ go‐go‐gene‐like (elk3) channel. Differential sensitivity for hERG1a and hERG1b.	diLQTs: reported at 10 μM attenuate electrophysiological effects associated with hERG inhibition by various drug inhibitors (e.g., high‐affinity E‐4031 dofetilide, low‐affinity moxifloxacin) but did not reverse the inhibition to the control levels.	Induces ventricular tachycardia and ventricular fibrillation in Langendorff‐perfused Guinea pig hearts at high concentrations, but not in rabbit hearts. Modifies vulnerable temporal window possibly via the effect on Nav1.5 channels.	16, 27, 32, 41, 48, 58, 61, 70, 84
VU0405601	2, 3	Likely to bind from outside to the ectodomain of the hERG channel.	No or small effect of 50 μM on recombinant cardiac Kv1.5 (I_Kur_), Nav1.5 (I_Na_), and KCNQ1 + KCNE1 (I_Ks_) channels.	diLQTS: reported to weaken interactions between hERG channel and dofetilide, astemizole, sertindole, and cisapride by an allosteric mechanism. Dofetilide‐induced arrhythmias were reduced after pretreatment with VU0405601	No report	19, 21

## 4 MOLECULAR DETERMINANTS OF hERG AGONIST ACTIONS ON THE *I*_Kr_ / *I*_hERG_ CHANNEL

Molecular determinants of the agonist activity of most hERG activators are yet to be elucidated, but a few studies have indicated the existence of multiple binding sites for agonist drug molecules on the hERG channel.^17^, ^19^, ^21^, ^22^, ^27^, ^42^, ^43^, ^44^, ^45^, ^46^, ^47^, ^48^, ^49^, ^50^, ^51^, ^52^, ^53^, ^54^ This is in contrast with hERG inhibitors which in their vast majority bind within the inner cavity of the channel.^2^, ^6^, ^37^, ^39^ The existence of multiple binding sites was first supported by the experimental observation that some hERG activators have a reported dual mode of action,^22^, ^23^, ^27^, ^44^, ^45^, ^46^, ^47^ acting as inhibitors at high concentrations.^22^, ^23^, ^27^, ^44^, ^45^, ^46^, ^47^ Structural and mutagenesis studies later identified several binding sites located on the intracellular side of the hERG channel, with residues located in S4 and S4–S5 linker^42^, ^44^, ^48^, ^49^, ^50^ or at sites overlapping that of hERG canonical drug inhibitor‐binding site.^55^ These studies also reported potential binding sites for hERG agonists on the extracellular side of the hERG channel near the selectivity filter.^42^, ^50^, ^51^, ^52^, ^53^, ^56^

The molecular mechanism(s) by which activators mediate their pharmacologic effects remain controversial.^39^, ^42^ They are thought to act mainly by modifying the hERG channel's gating properties and/or pharmacological sensitivity either directly or allosterically.^39^, ^42^ Thus, activators with binding sites overlapping that of the canonical binding site for high‐affinity block were suggested to directly compete with hERG inhibitors for binding within the inner cavity.^55^ In contrast, hERG activators with binding sites distinct from that of the hERG canonical binding site have been suggested to not compete with drug inhibitor molecules for binding onto the hERG channel for some^54^ or for others to produce negative allosteric modulation of hERG channel pharmacological sensitivity.^17^, ^19^, ^20^, ^39^ Agonist drug molecules reported to act as negative allosteric modulators of hERG high‐affinity pharmacological blockade were first identified in radioligand experiment assays (namely LUF7244, LUF7346, VU0405601, and MLT‐531).^19^ In this study, the incomplete dissociation of the high‐affinity inhibitor dofetilide from the hERG canonical binding site in the presence of LUF7244 indicated that the two drug molecules had nonoverlapping binding sites.^19^ However, LUF7244 has recently been suggested in a docking simulation into the hERG pore domain to bind below the selectivity filter within the inner cavity to a site that might overlap that of the hERG canonical binding site for high‐affinity block.^17^ This would suggest direct competition between LUF7244 and dofetilide drug molecules and conflict with radioligand experimental findings from the initial report.^19^ However, in the scheme of high‐affinity block of the hERG channel (see Section 2 in this review) and similar to previously reported data for activator PD118057,^52^ this could still be interpreted as an allosteric effect of LUF7244 that would modulate hERG pharmacological sensitivity to dofetilide, primarily via its ability in suppressing inactivation‐dependent conformational changes of the inner cavity a key determinant of hERG high‐affinity blockade (see Section 2). Although this is plausible, further investigations are warranted to support this scenario as data obtained from docking simulation of LUF7244 into hERG's pore inner cavity were not supported by data obtained from mutagenesis studies. The mechanism proposed on the basis of the data obtained from radioligand binding assays remains therefore to date the most likely mechanistic hypothesis for LUF7244 interactions with the hERG channel. Finally, it should be noted that the ability to allosterically reduce hERG inhibitors’ interactions with the channel's inner cavity may not be specific to hERG activators such as LUF7244, that is, exhibiting strong modulation of inactivation gating, but a shared characteristic with other types as suggested in functional or in silico experimental studies of the effects of type 1 LUF7346^19^, ^57^ and type 4 activator PD‐118057.^39^

## 5 ANTIARRHYTHMIC ACTIONS OF hERG AGONISTS

Antiarrhythmic benefits with the use of hERG activator drug molecules have been reported in several in vitro, in vivo, and in silico experimental models of cLQTS and/or diLQTS.^16^, ^17^, ^18^, ^19^, ^20^, ^21^, ^22^, ^23^, ^24^, ^25^, ^26^, ^27^, ^28^, ^29^, ^30^, ^31^, ^32^ However, these studies also revealed an increased risk of triggered activity and associated arrhythmia attached to their use in animal and in silico models.^16^, ^24^, ^30^, ^32^ It was initially suggested that the antiarrhythmic benefit of hERG agonists would be proportional to their potency in hindering the hERG's channel C‐type inactivation,^16^, ^21^, ^29^, ^30^, ^31^ with this effect mediating an increase in resurgent *I*_hERG_ (also see Section 2), thereby increasing the postrepolarization refractory period (PRRP). However, a risk of overcorrection of the APD to the point of triggering arrhythmia associated with the use of hERG activators exhibiting type 2 mechanism of actions has also been reported,^14^, ^16^, ^30^, ^58^ with this effect being concentration‐dependent.^16^, ^24^, ^30^ Thus, at high concentrations, the type 2 activator ICA‐105574 has been reported to cause, in various animal and human models, pharmacologically induced short QT syndrome (SQTS) and associated arrhythmia,^16^, ^24^, ^30^, ^58^ raising caution in its use. In contrast, type 1 activators, by mainly slowing down the rates of *I*_Kr_/*I*_hERG_ deactivation, may cause an increase in persistent *I*_Kr_ in early diastole thereby increasing cellular PRRP and counteracting potentially arrhythmogenic premature depolarizations (^18^, ^26^ see also Section 3 in this review). Consequently, their use has been suggested as a safer alternative to that of type 2 activators,^18^, ^26^ which through their mediated removal of inactivation alone have been associated with a high risk of overcorrection of the APD and associated arrhythmia.^16^, ^24^, ^30^, ^58^ Reports of the effects of agonist drug molecules exhibiting type 3 or 4 properties for the pharmacological management of diLQTS and cLQTS are by comparison scarce,^22^, ^59^ although they have been reported to increase the PRRP and shorten the QT interval but potentially to the point of triggering arrhythmia such as the type 3 hERG activator mallotoxin or type 4 PD118057.^21^, ^60^, ^61^ Given the limited investigations of the use of type 3 and 4 activators for the pharmacological management of LQTS, the next section will focus on a few examples of the most effective type 1 and 2 hERG agonists to date and review their reported antiarrhythmic benefits as well as associated pro‐arrhythmic risk for the management of LQTS.

## 6 hERG AGONIST DRUG CANDIDATES FOR THE PHARMACOLOGICAL MANAGEMENT OF LQTS

*The type 1 activator RPR260243* was the first discovered hERG agonist^23^ and shown to be effective against dofetilide‐induced arrhythmia in both guinea pig^23^ and zebrafish hearts.^18^ In the zebrafish heart, actions of the type I activator RPR260243 at 30 μM were associated with abbreviated APD, reduced triangulation of the AP, and increased refractory period through enhancement of hERG protective currents.^18^ An increase in the slope of the restitution curve was also observed and although reported as a potential antiarrhythmic benefit in this study this mechanism may carry a pro‐arrhythmic risk^18^; which raises caution. In a recent study from the same group, investigating the effects of RPR260243 on the fast‐deactivating congenital LQT2‐associated R56Q hERG mutant,^26^ RPR260243 was suggested to selectively increase hERG protective current in the early refractory period through its ability to reduce the hERG channel's rates of deactivation.^26^ Little to no increase in resurgent hERG amplitude during the AP was observed^26^; suggesting that the use of low concentrations of RPR260243 carries a limited risk of overcorrection of the APD while effectively protecting against triggered activity. This study may constitute a proof of concept of a potential mechanism‐based therapy for the management of LQT2‐associated hERG deactivation defective mutants (the main cause of LQT2 see Section 2) using RPR260243.

*The type 2 activator ICA‐105574* is the most potent activator to date and one of the first type 2 activators reported. It was first reported that concentrations below 5 μM (EC_50_ I_hERG_ 0.5 μM) prevent moxifloxacin‐induced ventricular tachycardia and fibrillation in Langendorff‐perfused guinea pig hearts,^16^ with this effect suggested to be mediated by its ability to completely reverse I_hERG_ inhibition by moxifloxacin to control levels.^16^ However, applied alone at the single concentration of 10 μM, and consistent with its reported strong modulation of I_Kr_ amplitude/kinetics,^16^, ^30^, ^45^ actions of ICA‐105574 were reported to cause an overcorrection of the APD^16^, ^30^, ^58^ to the point of triggering ventricular fibrillation.^16^, ^58^ The bisphenol NS1643, which also acts predominantly by reducing hERG channel inactivation but with modest potency compared with ICA‐105574,^16^, ^31^ was reported to exhibit a similar pro‐arrhythmic risk,^31^, ^58^ suggesting a strong link between pro‐arrhythmic risk and activator‐mediated attenuation of inactivation gating.^58^, ^60^, ^61^ The pro‐arrhythmic effect of ICA‐105574 was elucidated in a recent study examining the effects of this agent in a human‐induced pluripotent stem cell‐derived cardiomyocyte (hiPSC‐CM) model of cLQT2‐associated A422T hERG mutant.^24^ In this model, actions of ICA‐105574 at concentrations ≥3 μM were associated with an earlier peak in resurgent *I*_Kr_/*I*_hERG_ during the action potential of LQT2‐A422T expressing hiPSC‐CMs.^24^ This effect was suggested to cause early repolarization resulting in an overcorrection of the APD that in turn may shorten the QT interval to pro‐arrhythmic levels (SQT).^24^ Altogether, although ICA‐105574 had the ability to correct for the electrophysiological effects of mutant LQT2‐A422T, the risk of pharmacologically induced SQT associated with the use of concentrations as low as 3 μM^24^ raises concerns. Furthermore, across the ventricular wall, pro‐arrhythmic effects of ICA‐105574 have been suggested at higher concentrations to be associated with a significantly amplified transmural dispersion of repolarisation (TDR) and instability of the QT interval^16^, ^58^; both reported to be strong biomarkers of pro‐arrhythmic risk.^2^, ^58^ Similarly, NS1643 was reported to also markedly amplify these two biomarkers but only in the presence of an *I*_Ks_ inhibitor,^16^ which is consistent with its reported modest modulation of I_hERG_ kinetics compared with ICA‐105574 but also provides further evidence of the strong correlation between impaired inactivation gating and high pro‐arrhythmic risk.

*The type (1/2) activator LUF7244* shares structural similarities with the type 2 activator ICA‐105574^17^ and has been reported to act primarily through attenuation of rapid inactivation,^17^ although its effects have also been associated with significant slowing of the hERG channel's deactivation kinetics.^17^ Further investigations are therefore warranted before this pharmacological agent can be formerly categorised as a type 2 or mixed 1/2 activator. This agent has been reported to have a higher selectivity for hERG relative to other ion channels (LUF7244 at 10 μM had no effects on *I*_KIR2.1_, *I*_Nav1.5_, *I*_Ca‐L_, and *I*_Ks_ but doubled *I*_Kr_ ^17^). In contrast, RPR260243 at 30 μM exhibited weak inhibition of *I*_Ca‐L_ ^23^ which may underlie its effects on APD at this concentration. In a model of the canine atrioventricular block,^17^ 10 μM LUF7244 prevented dofetilide‐induced TdP arrhythmia in intact canine hearts. It failed, however, to return dofetilide‐induced APD prolongation to baseline in both hiPSC‐CMs and ventricular dog cardiomyocytes,^17^ indicating that the antiarrhythmic action of LUF7244 becomes apparent at lower concentrations than its effect against dofetilide‐induced AP prolongation observed at the single‐cell level.^17^ Altogether this suggests that (i) complete channel unblock may not be necessary to restore normal hERG activity and (ii) LUF7244 allosteric modulation of hERG pharmacological sensitivity may be secondary to the changes induced to the channel's gating properties,^17^ which is in accord with the proposed model of LUF7244‐mediated allosteric coupling between channel's gating and drug inhibitor interactions with hERG's inner cavity (see Section 4 in this review). Further experimental investigations are, however, required to highlight the exact underlying mechanism. Another potential use of great clinical relevance for LUF7244 was highlighted in a recent study showing that pretreatment with a combination of LUF7244 and the high affinity inhibitor dofetilide, but not LUF7244 alone, enhanced the membrane expression of both WT and trafficking defective LQT2‐associated G601S hERG mutant.^25^ Class III antiarrhythmic drugs such as dofetilide have been reported to rescue the expression of trafficking defective hERG channels but because of their *I*_Kr_/*I*_hERG_ inhibitory properties, this has been of little clinical relevance.^25^ The use of hERG agonist to mitigate the inhibitory effects of drug inhibitors that have been shown to be effective in rescuing defective channel trafficking, in both congenital and acquired forms of LQTS, may therefore constitute a new therapeutic tool.

*The type 1 activator LUF7346* was reported to activate *I*_hERG_ through the slowing of channel deactivation but unlike RPR260243 exhibits relatively significant attenuation of hERG inactivation gating^20^ however with no reported associated increase in pro‐arrhythmic risk.^20^ This may be in part due to its reported limited effects on other ion channels (*I*_Ks_, *I*_CaL_, *I*_Na_, and *I*_K1_)^20^ compared with RPR260243.^23^ More likely, this may be due to *LUF7346*6's possible prolonging effects of postrepolarization refractoriness which are yet to be thoroughly examined. LUF7346 effects have been comparatively less well studied with only one study showing that this agent can reverse the phenotype of isogenic pairs of iPSC cells containing the LQT2‐associated c.A2987T (N996I) *KCNH2* mutation,^20^ while also able to rescue channel function following drug‐induced QT prolongation in this same model.^20^ A similar strategy to increase *I*_hERG_ elicited from LQT2‐associated hERG trafficking‐defective mutants was previously investigated for the type 1,2,3 NS1643 [Correction added on June 22, 2022, after first Online publication: In the sentence 'A similar strategy …', type 2 has been changed to type 1, 2, 3.],^62^ type 2 ICA‐105574,^28^ type 4 PD‐118057,^59^ and ITP‐2^28^ but with mixed results (^28^, ^59^ see also Tables 1, 2, 3 in “Reported benefit for LQTS”), suggesting that LUF7346 combined with its suggested effect on postrepolarization refractoriness would be of superior antiarrhythmic benefit for the management of cLQTS. Also of clinical value in this study are the reported recapitulating effects of LUF7346 on an LQT1 and a Jervell and Lange Nielsen (JLNS)‐associated KCNQ1 mutant channels (^20^; see also Table 1 “Reported benefit for LQTS”), suggesting that LUF7346 may also be of use for the management of other forms of cLQTS. Similar positive effects in hiPSCMs harboring JLNS‐associated KCNQ1 mutations were reported for the type 1,2 activator MLT‐531^29^ and activator with multiple mechanisms of actions (types 1, 2, 3) NS1643,^63^ with effects of both activators suggested to be mainly mediated through their modulation of the hERG's channel inactivation but not deactivation gating. Altogether, this would suggest that LUF7346 with its combined effects on APD and postrepolarization refractoriness through its modulation of hERG's deactivation gating may provide superior antiarrhythmic benefit to that of NS1643 or MLT‐531 for the management of the effects associated with JLNS.

## 7 LIMITATIONS OF THE PROPOSED STRATEGY

Although the selective activation of *I*_Kr_/*I*_hERG_ by its drug agonist molecules appears to be a promising strategy for the pharmacological management of LQTS, there are still many potential drawbacks to address before these results can be validated in clinical studies. First, a growing body of experimental evidence suggests that hERG agonist drug molecules may exhibit narrow therapeutic windows that may be attributable, in part, to the reported dual mode of action of hERG activators acting as blockers at high concentrations^22^, ^23^, ^27^, ^44^, ^45^, ^46^, ^47^ and in part to the selectivity hERG activators exhibit for hERG relative to other ion channels (see row “Channel selectivity” in Tables 1, 2, 3). Thus, the agonist drug molecule NS1643 with reported mixed type 1, 2, and possibly 3 properties was shown at low concentration to be effective against triggered activity in in vitro and ex vivo experimental models but was later revealed by in silico modeling of the human cardiomyocyte to enlarge the vulnerable window for the development of reentry circuits thereby increasing pro‐arrhythmic risk.^32^ This effect was suggested to be related to NS1643's inhibitory effects against Nav1.5 channels.^32^ A similar effect was reported for the activator MC‐II‐157c an analog of NS1643 with enhanced hERG agonist activity compared with NS1643,^64^ suggesting that widening the window for agonist activity does not overrule inhibitory effects against Nav1.5 and associated pro‐arrhythmic risk. Attempts to rationally design derivatives of hERG activator drug molecules to turn these compounds into full agonist drug molecules of the hERG channel have failed,^48^, ^57^ with limited effect and reported loss of agonist activity for derivatives of NS1643 and LUF7346.^48^, ^57^ Altogether, this suggests that the iterative design of hERG activators to enhance their therapeutic window may have limited applications.

Another critical factor that may underlie narrow therapeutic windows is the increased pro‐arrhythmic risk associated with the strong modulation of the hERG's channel gating properties, mainly inactivation, by some hERG agonist drug molecules (see Sections 5 and 6 in this review). Further, at high concentrations, some hERG agonists have been shown to impact negatively on biomarkers of the arrhythmogenic substrate (e.g., TDR and/or QT instability^16^, ^58^, ^60^, ^61^). This is critical as pharmacological strategies for the management of LQTS that had only aimed to modulate positively the triggers of arrhythmia have shown limitations.^32^, ^58^, ^60^, ^61^, ^64^ All together this should be considered in future studies aiming to validate hERG agonist as drug candidates for the pharmacological management of LQTS. Finally, it has been reported that hERG agonist NS3623 impaired cardiac conduction in Langendorff‐perfused guinea pig hearts.^65^ This agent has also been suggested to induce adverse activation of *I*_Kr_ in both the sinus node and vagal fibers,^66^ where *I*_Kr_/*I*_hERG_ participates in the control of the heart rhythm.^6^ Modulation of the pacemaking activity in the sinus node by native *I*_Kr_ has been suggested to be underlined by a slow decay in persistent diastolic *I*_Kr_ (see Sections 2 and 5 in this review) that would favor the firing of a new AP (67also reviewed in 6), with inhibition of *I*_Kr_ reported to be associated with slowing of SA node firing.^6^ A similar role has been reported for *I*_Kr_ in rabbit atrioventricular (AV) nodal cells,^68^ suggesting that *I*_Kr_ may also participate in the auriculo‐ventricular conduction. In that context, it could be speculated that hERG agonist‐mediated increase in *I*_Kr_/*I*_hERG_, and type 1 activators in particular for their mediated increase in persistent diastolic *I*_Kr_, may modify both pacemaking activity of the SA node and conduction through the AV node. These unintended potential effects of hERG agonists on both cardiac conduction and pacemaking activity could be critical because heart rate and length of the QT interval are correlated with reported greater QT prolongation at slow heart rates. Altogether this highlights the need for further investigations.

## 8 PERSPECTIVES

Although some hERG agonist drug molecules may constitute promising drug candidates, there are still extensive preclinical studies in animal and/or in silico models to carry out before these drugs can be proposed for early stages of clinical trials; including preliminary studies of their efficacy, toxicity, pharmacokinetics, and safety information. Thus, knowledge is lacking on the effects associated with the long‐term use of hERG agonist drug molecules on both cardiac and noncardiac tissues. Kv11.1 channels are widely distributed in various organs and *I*_Kr_ takes part in many biological processes.^6^ Some hERG activators have already been reported to exhibit a differential effect against noncardiac (neuronal) isoforms of the hERG channel and/or other members of the “erg” channel family (see Tables 1, 2, 3, row “channels selectivity”). This would be suggestive of a possible differential effect of *I*_Kr_ activation in noncardiac tissues. However, this remains hypothetical as there is to date no information available on hERG activator drug molecules transport across the blood–brain barrier and/or whether their effects would be limited to the peripheral circulation. Also of importance is the reported interspecies variation in the response of *I*_Kr_ to hERG agonist actions (discussed in 17, 18, 58). This is likely due to the interspecies variability in the relative expression of ionic channels and related APD reliance on *I*_Kr_ with some cardiac animal models suggested to be more susceptible to the role of *I*_Kr_ in the development of EADs/mechanism of arrhythmia.^69^ It could also be related to the suggested relative importance of the isoform hERG1b in human repolarization as opposed to other species.^6^, ^33^ To date, only two studies have suggested differential sensitivity to hERG agonist actions of heteromeric channels hERG1a/1b compared with homomeric hERG1a channels;^28^, ^70^ highlighting that our understanding of hERG agonist exact actions against native cardiac *I*_Kr_ is rather limited. All together this warrants mandatory investigations in human relevant studies for the full characterization of the effects of hERG activators on human ventricular repolarization and of their adverse toxicity.

A first step toward better translational characterization of the effects of hERG agonist would be to comprehensively assess their cardiac effects in line with the recommendations of the Comprehensive in Vitro Pro‐arrhythmia Assay (CiPA) initiative.^71^ This initiative was first established to develop a new paradigm for assessing pro‐arrhythmic risk associated with the use of drugs in development and expand our understanding of torsadogenic mechanisms beyond hERG pharmacological blockade. It advocates for the generalized assessment of a drug's effect on multiple ion channels, integration of these effects in a computer model of the human cardiomyocyte to predict pro‐arrhythmic risk as well as the use of fully integrated biological systems with human stem cell‐derived cardiomyocytes, and ECG analysis in early phase I clinical trials.^71^ Although some studies have partially addressed some of the CiPA recommendations, this strategy needs to be generalized as it could offer valuable insights into the safety of the use of hERG agonists for the management of both congenital and acquired LQTS.

Also, of importance for future evaluations is the clinical relevance of experimental conditions. In fact, there has been limited attention to whether experimental conditions are representative of common clinical settings where more than one QT‐prolonging factor may occur concomitantly, generating an amplifying effect.^2^, ^7^ The question of how the actions of hERG activators will react to concomitant presence of one or more drugs (polypharmacy), a genetic predisposition (polymorphism), underlying cardiac condition such as myocardial infarction or a combination of all these aggravating factors as seen in elderly patients^7^ still needs to be comprehensively addressed.

## 9 CONCLUSION

Since the discovery of the first hERG activator in 2005, it has now emerged that type 1 hERG activators, as opposed to other types of hERG agonist (particularly type 2 with their strong modulation of inactivation gating and related increased pro‐arrhythmic risk), may constitute good candidates for the pharmacological management of congenital and drug‐induced LQTS. Their progress as therapeutic agents, however, still lacks critical preclinical knowledge of their actions.

In parallel, new pharmacological therapies for the management of LQTS are emerging. Thus, the use of late sodium current blockers such as mexiletine, ranolazine, and/or lidocaine have been suggested for the management of cLQTS,^3^ but also diLQTS^13^, ^15^ especially in cases refractory to conventional interventions where removal of culprit drug and or ICD implantation are undesirable or contraindicated.^13^ Mechanism‐based therapies for the management of cLQTS have also been explored. Thus, in patients with hERG trafficking defects, lumacaftor, a drug that was shown to restore intracellular trafficking of mutated protein products such as CFTR, has been shown to shorten the QT interval of LQT2 patients significantly.^72^ Further investigations are, however, warranted as this study was very limited in terms of the number of patients included.^72^ Further, the use of siRNA^5^ or CRISPR/Cas9 gene‐editing technology^5^ for the management of cLQT2 constitutes potential new avenues of treatment. All these strategies, however, also have limitations of their own. A comprehensive comparison of the balance between the benefits and risks associated with their use compared with that of type 1 hERG activator drug molecules may be useful and constitute a great advancement in the field.

## CONFLICT OF INTEREST

All authors declare that they have no conflict of interest related to this review.
